# Iron partitioning at an early growth stage impacts iron deficiency responses in soybean plants (*Glycine max* L.)

**DOI:** 10.3389/fpls.2015.00325

**Published:** 2015-05-12

**Authors:** Carla S. Santos, Mariana Roriz, Susana M. P. Carvalho, Marta W. Vasconcelos

**Affiliations:** ^1^Centro de Biotecnologia e Química Fina – Laboratório Associado, Escola Superior de Biotecnologia, Universidade Católica PortuguesaPorto, Portugal; ^2^Horticultural and Product Physiology Group, Department of Plant Sciences, Wageningen UniversityWageningen, Netherlands; ^3^Faculty of Sciences, University of PortoPorto, Portugal

**Keywords:** soybean, partitioning, iron deficiency chlorosis (IDC), IRT1, FRO2, ferritin

## Abstract

Iron (Fe) deficiency chlorosis (IDC) leads to leaf yellowing, stunted growth and drastic yield losses. Plants have been differentiated into ‘Fe-efficient’ (EF) if they resist to IDC and ‘Fe-inefficient’ (IN) if they do not, but the reasons for this contrasting efficiency remain elusive. We grew EF and IN soybean plants under Fe deficient and Fe sufficient conditions and evaluated if gene expression and the ability to partition Fe could be related to IDC efficiency. At an early growth stage, Fe-efficiency was associated with higher chlorophyll content, but Fe reductase activity was low under Fe-deficiency for EF and IN plants. The removal of the unifoliate leaves alleviated IDC symptoms, increased shoot:root ratio, and trifoliate leaf area. EF plants were able to translocate Fe to the aboveground plant organs, whereas the IN plants accumulated more Fe in the roots. *FRO2*-like gene expression was low in the roots; *IRT1*-like expression was higher in the shoots; and *ferritin* was highly expressed in the roots of the IN plants. The efficiency trait is linked to Fe partitioning and the up-regulation of Fe-storage related genes could interfere with this key process. This work provides new insights into the importance of mineral partitioning among different plant organs at an early growth stage.

## Introduction

Soybean (*Glycine max* L.) is the highest produced legume crop, reaching production levels of about 230 million metric tons per year, across the world (Vasconcelos and Grusak, 2014). In many agricultural areas, where calcareous soils are predominant, iron (Fe) availability becomes a yield-limiting factor with major economic implications for field crop production (Rodríguez-Lucena et al., 2010). Since Fe is an essential element that has a key role in fundamental biological processes, such as photosynthesis and chlorophyll biosynthesis, when this micronutrient is unavailable to the plants, they frequently exhibit yellowing of the upper leaves, interveinal chlorosis, and stunted growth (Jeong and Connolly, 2009). This problem underpins the urgency to develop cultivars that can be more efficient in Fe uptake and further mineral translocation from the roots to the shoots, thus increasing plant nutritional value (Carvalho and Vasconcelos, 2013).

For a long time, soybean plants have been differentiated between EF, if they respond to Fe-deficiency stress by inducing biochemical reactions that make Fe available in a useful form, and IN if they do not (Brown, 1978; García-Mina et al., 2013). However, there is scarce information about the physiological and molecular mechanisms behind tolerance to iron deficiency and about the mechanisms that govern the partitioning of captured mineral nutrients between different plant organs (Vasconcelos et al., 2006; Lemoine et al., 2013; Roriz et al., 2014).

Plants have been divided between Strategy I and Strategy II, depending on their mechanism for Fe uptake. Dicoteledonous and non-grass monocoteledonous plants depend on an Fe reduction mechanism that allows them to reduce Fe (III) to Fe (II) in the rhizosphere (Abadía et al., 2011). Whilst the first Fe form is the most abundant in soils, it is poorly soluble at neutral or basic pH and, therefore, unavailable for uptake, causing IDC. A plasma-membrane Fe(III)-reductase, encoded by the *FRO* gene family, favors inorganic Fe solubilisation and consequent uptake by an Fe(II)-transporter, IRT1, of the ZIP family (Moog and Brüggemann, 1994). On the other hand, when in need for Fe accumulation and storage, plants augment the expression of ferritin, which plays a role in buffering excess Fe in plants (Roschzttardtz et al., 2013). However, excess accumulation in the form of ferritin can impair Fe remobilization from one plant organ to another (Vasconcelos and Grusak, 2014).

The regulation of sink-source relations is a complex process (Fester et al., 2013). It is well-known that mineral nutrient deficiencies may substantially influence dry matter partitioning between plant organs (Marschner et al., 1996), as nutrient-deprived plants generally tend to invest in their root system (Lemoine et al., 2013). Moreover, the shoot to root communication may act as an important feedback control signal for nutrient uptake and partitioning. For instance, sufficient Fe content in the leaves can modulate the synthesis of the ferric chelate reduction system and the capacity of the phloem to carry Fe from the roots, regulating the ‘EF reaction,’ acting as a negative feedback control (Maas et al., 1988).

Source leaves export photoassimilates to sink tissues when the demand exceeds the production via photosynthesis (Ludewig and Flügge, 2013) and nutrient movement to sink tissues could be controlled by the dynamics of source-sink carbohydrate partitioning (Grusak, 2002). Besides, the sink–sink competition also influences these regulatory processes, usually with one plant organ having a negative effect upon another by consuming or controlling access to a resource that is limited in its availability (Sadras and Denison, 2009). Hence, nutrient deficiency may not only affect the provision of photosynthates by decreasing source capacity, but also by altering partitioning between the source organs and various sinks (Marschner et al., 1996). Therefore, studies on Fe deficiency have utilized leaf excision to better understand the mechanisms of long-distance signaling. For example, the removal of leaves gave positive insights about the regulation of the *NtIRT1* and *NtFRO1* expression in roots of Fe-deficient tobacco plants (Enomoto et al., 2007). In another study, the shoot-tip was removed from apple plants, to understand the role of hormones in the regulation of Fe deficiency responses (Wu et al., 2012). Both studies found that shoots play a critical role in regulating Fe uptake in roots. To the best of our knowledge, few studies have focused on the role of nutrient competition between sink organs in the Fe deficiency responses of contrasting cultivars. So far, studies on efficiency have focused on identifying genetic markers for use in breeding programs, solely explaining the efficiency mechanism using genetic models (Lin et al., 1997; O’Rourke et al., 2007; Peiffer et al., 2012) and recent findings show that efficient genotypes induce energy controlling pathways to promote IDC resistance responses (Atwood et al., 2014). However, these studies only correlate the molecular results with the activity of the ferric chelate reductase or with chlorosis development. Therefore, there is a need for a study that integrates several possible traits that contribute for the efficiency mechanism in soybean plants.

The aim of this work was to understand if the ability to partition Fe could be related to IDC efficiency and to investigate the role of the expression of Fe uptake and storage related genes in this process. Given the fundamental importance of source/sink relations for plant growth and development, and that sink organs compete with each other for the carbohydrates and nutrients provided by source organs, we hypothesize that the ability to manage nutrient partitioning among different organs is an important trait contributing to an EF response. To verify this hypothesis, we removed the unifoliate leaves – strong sink organs in the early stages of plant development that have previously been shown to be correlated with IDC tolerance (Vasconcelos and Grusak, 2014) – and analyzed morphological, physiological, and molecular indicators in two *G. max* accessions with contrasting efficiencies for Fe-deficiency.

## Materials and Methods

### Plant Material, Growth Conditions, and Treatments

An efficient (EF – PI437929/VIR 316) and an inefficient (IN – PI378676A/Primorskaja 500) *G. max* accession for Fe deficiency (Vasconcelos and Grusak, 2014), with identical phenology, were selected from the USDA (United States Department of Agriculture) germplasm collection via GRIN (Germplasm Resources Information Network)^1^. Seeds were rolled in filter paper and placed vertically in a solution of 250 mM CaCl_2_, for 7 days in the dark, at 25°C. In the current work, plants were grown hydroponically mimicking Fe deficient soil. Studies have shown that similar QTLs associated with IDC are identified in nutrient solution and field tests and, therefore, both systems identify similar genetic mechanisms of iron uptake and/or utilization (Lin et al., 1998).

In Experiment 1 germinated seedlings were transferred to 20 L vessels containing hydroponic solution with different Fe treatments. Each vessel contained five plants of one accession grown in Fe sufficient (+Fe, 20 μM Fe(III)-EDDHA [ethylenediamine-*N*,N′bis(*o*-hydroxyphenyl)acetic acid]) or in Fe deficient [-Fe, 0 μM Fe(III)-EDDHA] conditions.

The vessels were placed in a climate chamber (Aralab Fitoclima 10000EHF) with 16 h day photoperiod providing 325 μmol s^-1^ m^-2^ of photosynthetic photon flux density at plant level supplied by a mixture of incandescent bulbs and fluorescent lights. Temperatures were set to 25°C during the light period and to 20°C during the dark period, whereas relative humidity was maintained at 75% throughout day and night. The standard solution for hydroponic growth of *G. max* included: 1.2 mM KNO_3_, 0.8 mM Ca(NO_3_)_2_, 0.3 mM MgSO_4_.7H_2_O, 0.2 mM NH_4_H_2_PO_4_, 25 μM CaCl_2_, 25 μM H_3_BO_3_, 0.5 μM MnSO_4_, 2 μM ZnSO_4_.H_2_O, 0.5 μM CuSO_4_.H_2_O, 0.5 μM MoO_3_, 0.1 μM NiSO_4_. Hydroponic solution was buffered with the addition of 1 mM MES [2-(*N*-morpholino)ethanesulfonic acid], pH 5.5 and, during the experimental time, pH was measured and solutions were changed weekly. The experiment ended 10 days after transferring the plants to the climate chamber.

**FIGURE 1:**
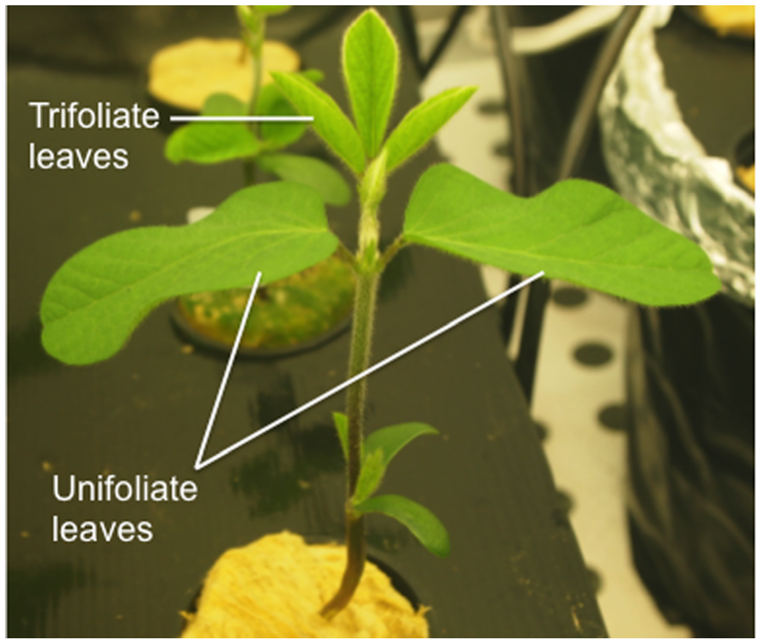
**Efficient accession soybean plant 437929 at V1 stage of development (as described by Fehr and Caviness, 1977) showing fully expanded unifoliate leaves and one unfolded trifoliate**.

To infer if the removal of the unifoliate leaves could alleviate IDC stress symptoms, a separate experiment was conducted (Experiment 2). In this experiment, plants were grown under the same conditions as described above, but unifoliate leaves were removed 3 days after the transfer to the hydroponic solutions (corresponding to about 10 mm length) and in the control plants the unifoliate leaves were kept on the plant. Please see **Figure 1** for the *G. max* anatomy visualization.

When the first unfolded trifoliate leaves of the inefficient accession showed signs of chlorosis, the experiments were terminated and plants were sampled for further analysis, which corresponded to 10 days after transferring the plants to the climate chamber.

### Morphological Parameters

Chlorosis scoring was conducted using a visual scale according to Wang et al. (2008): (1) no chlorosis, plants normal and green; (2) slight yellowing of the upper leaves, no differentiation in color between the leaf veins and interveinal areas; (3) interveinal chlorosis (green veins and chlorotic interveinal areas) in the upper leaves, but no obvious stunting of growth or death of leaf tissue (necrosis); (4) interveinal chlorosis of the upper leaves with some apparent stunting of growth or necrosis of plant tissue; and (5) severe chlorosis with stunted growth and necrosis in the youngest leaves. Also, Soil and Plant Analyzer Development (SPAD) readings were conducted with a portable chlorophyll meter (Konica Minolta SPAD-502Plus; Minolta, Osaka, Japan) at the end of 10 days, using the first expanded trifoliate leaf from the top of the plant.

Sampled roots, stems and leaves were separated, weighed, and measured for length. The material was then dried at 70°C until constant weight and stored for ICP-OES analysis. Foliar area of the trifoliate leaves was measured using a leaf area meter AM300 (ADC BioScientific Ltd., UK).

### Root Iron Reductase Activity Measurements

Root iron reductase was quantified as described by Vasconcelos et al. (2006). The measurements were carried out in roots of intact plants via the spectrophotometric determination of Fe^2+^ chelated to BPDS (bathophenanthroline disulfonic acid). Roots of each plant were submerged in assay solution containing: 1.5 mM KNO_3_, 1 mM Ca(NO_3_)_2_, 3.75 mM NH_4_H_2_PO_4_, 0.25 mM MgSO_4_, 25 μM CaCl_2_, 25 μM H_3_BO_3_, 2 μM MnSO_4_, 2 μM ZnSO_4_, 0.5 μM CuSO_4_, 0.5 μM H_2_MoO_4_, 0.1 μM NiSO_4_, 100 μM Fe(III)-EDTA (ethylenediaminetetraacetic acid) and 100 μM BPDS. All nutrients were buffered with 1 mM MES, pH 5.5. The assays were conducted under dim light conditions at 20°C and were terminated after 45 min by removal of the roots from the assay solution. Absorbance values were obtained spectrophotometrically at 535 nm, and an aliquot of the solution that had no roots during the assay was used as blank. Rates of reduction were determined using the molar extinction coefficient of 22.14 mM^-1^cm^-1^.

### Total Fe Determination by ICP-OES

One hundred mg of the dried plant tissues (root, stem, cotyledon, unifoliate, and trifoliate leaves) of the two *G. max* accessions grown as described above were mixed with 5 mL of 65% HNO_3_ in a Teflon reaction vessel and heated in a SpeedwaveTM MWS-3+ (Berghof, Germany) microwave system. Each plant organ from all the treatments (*n* = 5) was ground and five independent digestions were carried out.

Digestion procedure was conducted in five steps, consisting of different temperature and time sets: 130°C/10 min, 160°C/15 min, 170°C/12 min, 100°C/7 min, and 100°C/3 min. The resulting clear solutions of the digestion procedure were then brought to 20 mL with ultrapure water for further analysis. Mineral concentration determination was performed using the ICP-OES Optima 7000 DV (PerkinElmer, USA) with radial configuration.

### Gene Expression Analysis

Additional plants were grown under the conditions described above, collected at the end of the assay and immediately frozen in liquid nitrogen. A pool of five biological replicates from each treatment were grinded thoroughly with a mortar and pestle until a fine powder was obtained and total RNA was extracted using a Qiagen RNeasy Plant Mini Kit (USA, Nr. #74904), according to the manufacturer’s instructions. RNA quality and quantity were checked by UV-spectrophotometry, using a nanophotometer (Implen, Isaza, Portugal). Single-stranded cDNA was then synthesized using the First Strand cDNA Synthesis Kit (Fermentas UAB, Cat. Nr. #K1612) in a Thermal cycler (VWR, Doppio, Belgium), according to manufacturer’s instructions.

Sequence homologs to *AtFRO2* and *AtIRT1* in *G. max* were queried in NCBI database and the sequences with highest homology were selected (Supplementary Table S1). Primers for *FRO2*-like, *IRT1*-like, and *ferritin* were designed using Primer3^2^, specifying an expected PCR product of 100–200 bp and primer annealing temperatures between 56 and 58°C (Supplementary Table S2). qPCR reactions were performed on a Chromo4 thermocycler (Bio-Rad, Hercules, CA, USA) with the following reaction conditions: 10 min at 95°C and 40 cycles with 15 s at 95°C, 15 s at 58°C, and 15 s at 68°C. Amplifications were carried out using 1.25 μM of the specific primers and mixed to 12.5 μM of 2xPCR iQ SYBR Green Supermix (Bio-Rad) and 100 ng of cDNA in a final volume of 25 μl. Three technical replicates were performed for each gene tested in qPCR reactions, as well as for controls. Melt curve profiles were analyzed for each tested gene. The comparative CT method (ΔΔCT; Livak and Schmittgen, 2001) was used for the relative quantification of gene expression values of Fe related genes using the 18S rRNA gene as the control transcript (Opticon Monitor 3 Software, Bio-Rad) and the EF plants, grown under Fe sufficiency, with unifoliate leaves as the reference sample. Data were transferred to Excel files and plotted as histograms of normalized fold expression of target genes.

A heatmap with folds of expression was designed using R software (R Development Core Team, 2013).

### Statistical Analysis

Data were analyzed with GraphPad Prism version 6.00 for Mac OS X (GraphPad Software, La Jolla, CA, USA^3^). Differences between treatments were tested with unpaired Student’s *t*-test corrected for multiple comparisons using Holm-Sidak method. Statistical significance was considered at *P* < 0.05.

## Results

### IDC Symptom Evaluation and Reductase Activity Quantification

**FIGURE 2:**
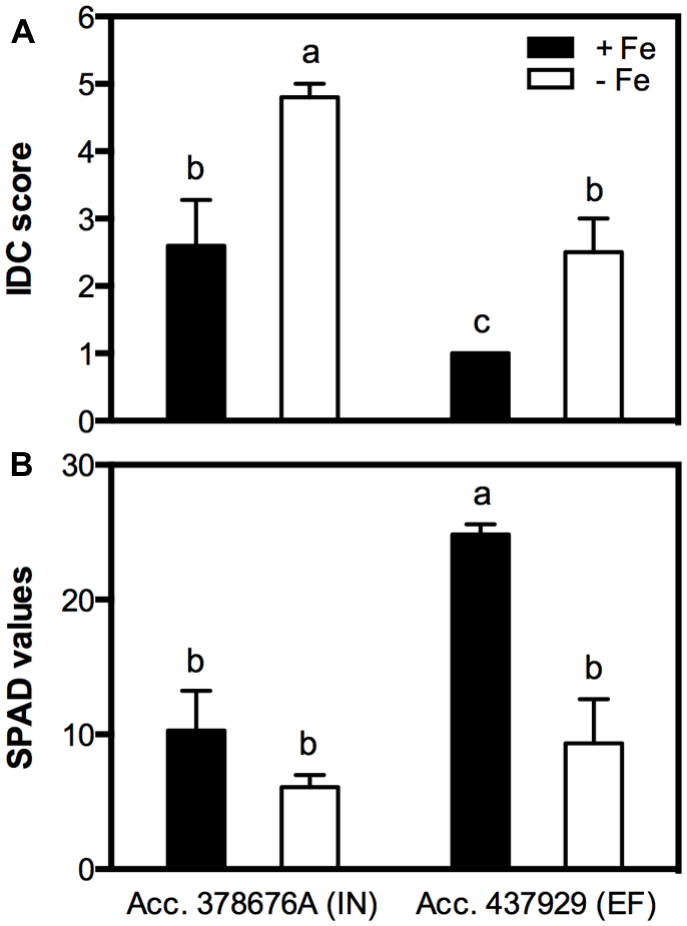
**Iron deficiency chlorosis (IDC) visual score **(A)** and Soil and Plant Analyzer Development (SPAD) values **(B)** in two *Glycine max* accessions [378676A (inefficient) and 437929 (efficient)] grown in Fe-sufficient (+Fe) and Fe-deficient (-Fe) hydroponic conditions.** Data are the mean ± SE of five biological replicates. Different letters indicate significant differences (*P* < 0.05).

The clearest symptom of IDC in plants is the interveinal yellowing of the younger leaves that can be assessed by using a visual chlorosis score in which 5 represents full chlorosis and 1, no chlorosis. EF plants grown in Fe sufficiency remained green throughout the experiment, while the IN ones presented some signs of chlorosis. The IDC visual scores of plants grown under Fe shortage were 4.8 ± 0.2 for the IN accession and 2.5 ± 0.5 for the EF one (**Figure 2A**). IDC was also evaluated measuring the chlorophyll content in the younger trifoliate leaves using a SPAD meter (**Figure 2B**). Average SPAD values corroborated that, when in -Fe conditions, the IN accession presented lower SPAD values (6.1 ± 0.9) than the EF one (9.3 ± 2.9), and even under Fe sufficiency the IN plants showed signs of chlorosis, with no significant differences to the -Fe plants (10.3 ± 3.0). The EF plants under Fe sufficiency had the highest SPAD values (24.9 ± 1.9).

**FIGURE 3:**
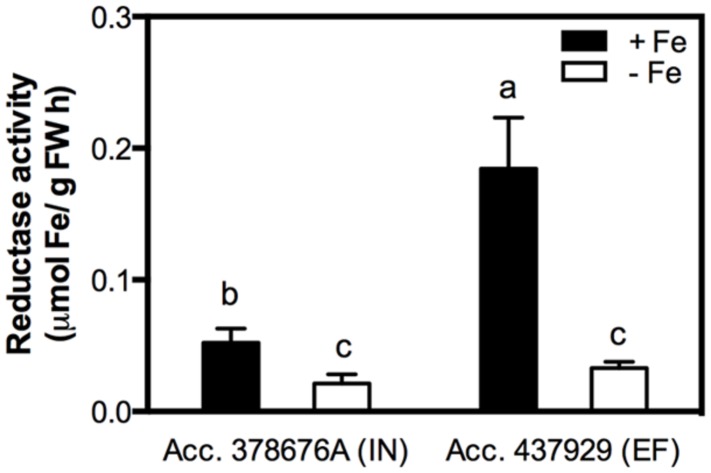
**Root reductase activity of two *G. max* accessions [378676A (inefficient) and 437929 (efficient)] grown in Fe-sufficient (+Fe) and Fe-deficient (-Fe) hydroponic conditions.** Data are mean ± SE of five biological replicates. Different letters indicate significant differences (*P* < 0.05).

Reductase activity was measured in roots of both IN and EF *G. max* accessions, grown under Fe shortage and Fe sufficiency. The Fe^3+^ chelate reductase activity was largely enhanced under Fe sufficiency for both accessions (**Figure 3**), and the activity of this enzyme was threefold higher in the EF accession.

### Effects of Unifoliate Leaf Removal on IDC Symptoms and Fe Partitioning

**FIGURE 4:**
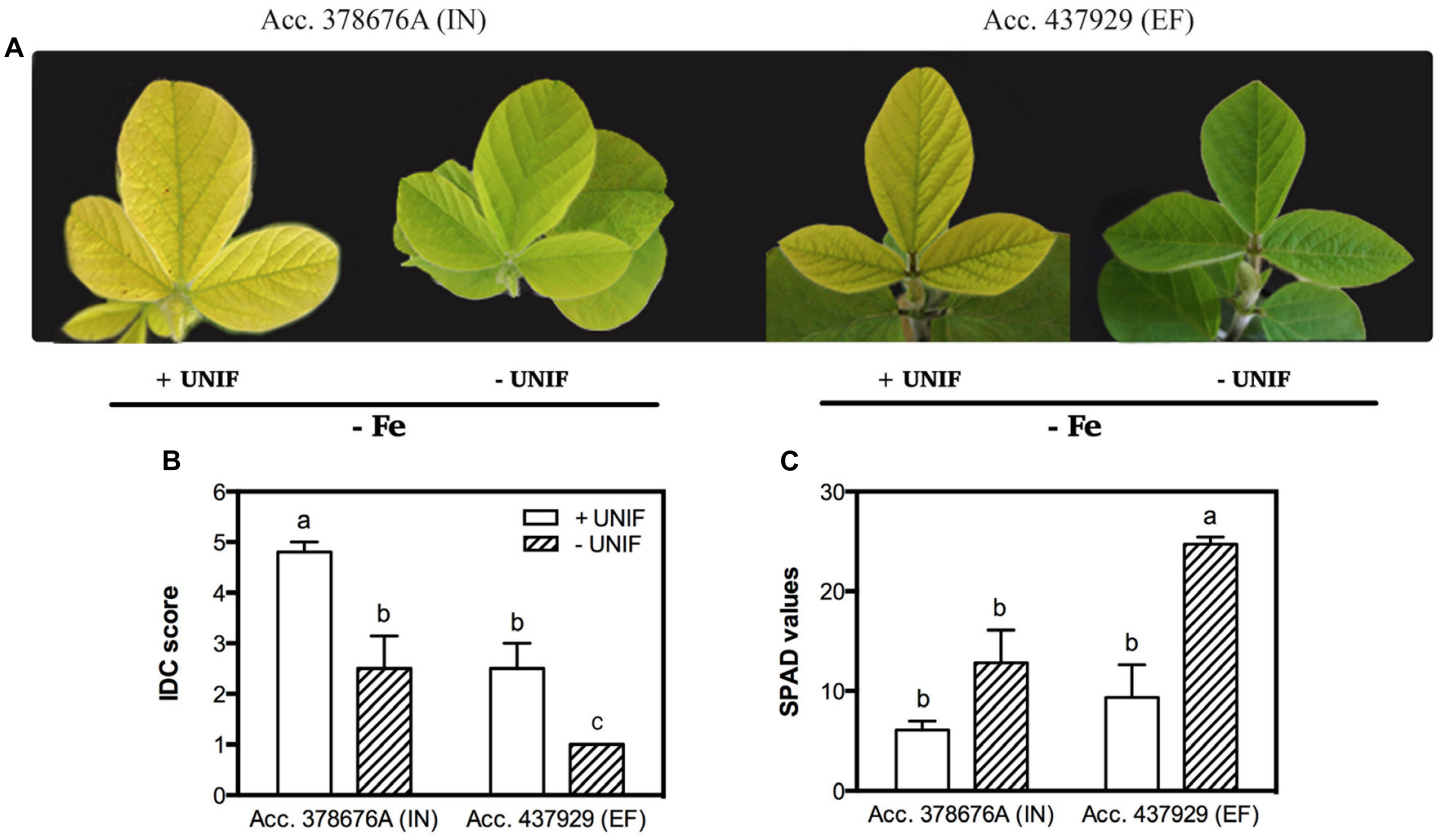
**Iron deficiency chlorosis visual symptoms **(A)**, visual IDC score **(B)** and SPAD values **(C)** in two *G. max* accessions [378676A (inefficient) and 437929 (efficient)] grown in Fe-deficient (-Fe) hydroponic conditions, with (+UNIF) and without (-UNIF) unifoliate leaves (Experiment 2). Data are mean ± SE of five biological replicates.** Different letters indicate significant differences (*P* < 0.05).

The effect of Fe partitioning on IDC responses was assessed by growing the two accessions and removing the unifoliate leaves at an early growth stage, and comparing these to intact plants. Under Fe sufficiency, unifoliate removal did not significantly impact IDC score, SPAD values, plant DW and trifoliate leaf area (data not shown). Under Fe deficiency, intact plants presented accentuated visual symptoms of chlorosis, particularly in the IN accession (**Figure 4**).

**FIGURE 5:**
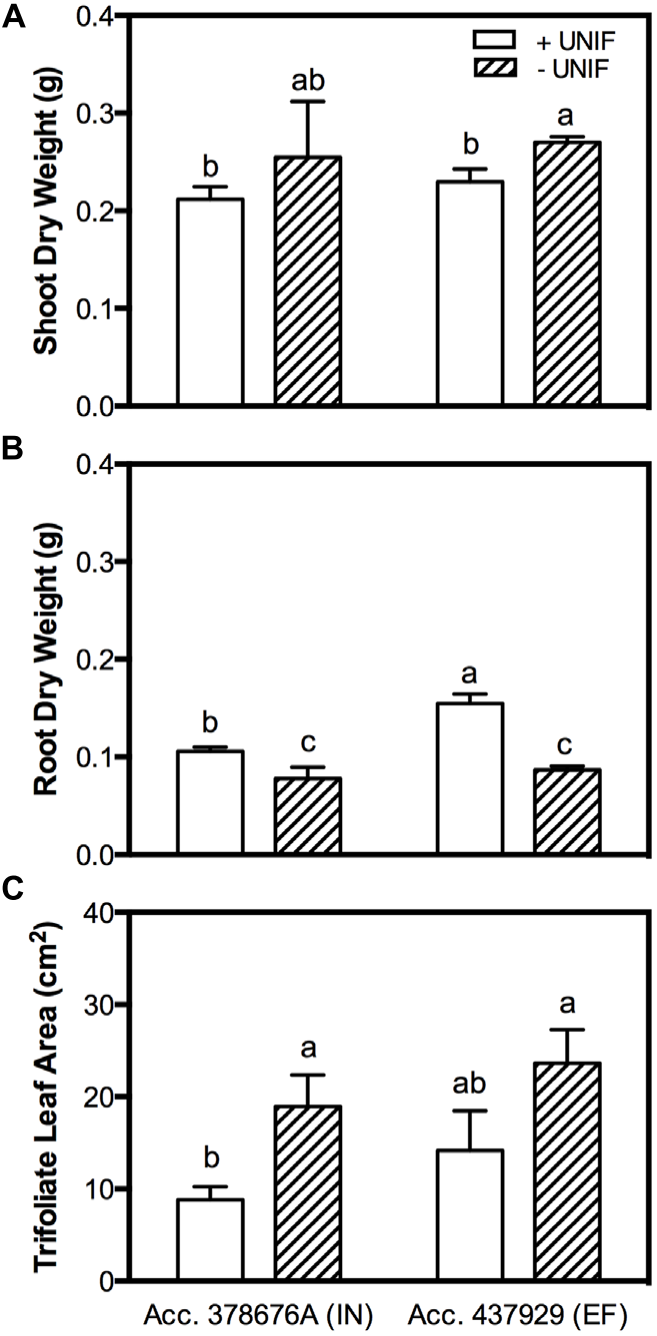
**Shoot DW (accounting the unifoliate leaves weight; **(A)**, root DW **(B)** and trifoliate leaf area **(C)** of two *G. max* accessions [378676A (inefficient) and 437929 (efficient)] grown under Fe-deficient (-Fe) hydroponic conditions, with (+UNIF) and without (-UNIF) unifoliate leaves (Experiment 2).** Data are mean ± SE of five biological replicates. Different letters indicate significant differences (*P* < 0.05).

The removal of the unifoliate leaves (-UNIF) led to significant improvements in the IDC symptoms, in both accessions (**Figures 4** and **5**). For instance, the IN accession presented a reduction of IDC visual score from 4.8 to 2.5 (**Figure 4B**) and the SPAD values were increased by 53% (**Figure 4C**). In the EF plants, the IDC visual score was significantly reduced from 2.5 to 1 and the SPAD values increased 62%. Moreover, the removal of the unifoliate leaves led to an increase in the shoot DW in both accessions but this was only significantly higher in the EF plants (**Figure 5A**). On the other hand, root DW was significantly lower in plants without unifoliate leaves, representing a decrease of 27% for the IN plants and 44% for the EF plants (**Figure 5B**). Finally, IN plants grown without unifoliate leaves presented 53% larger trifoliate leaf area and the EF plants had a 40% increase although in the last case this difference was not significant (**Figure 5C**).

**FIGURE 6:**
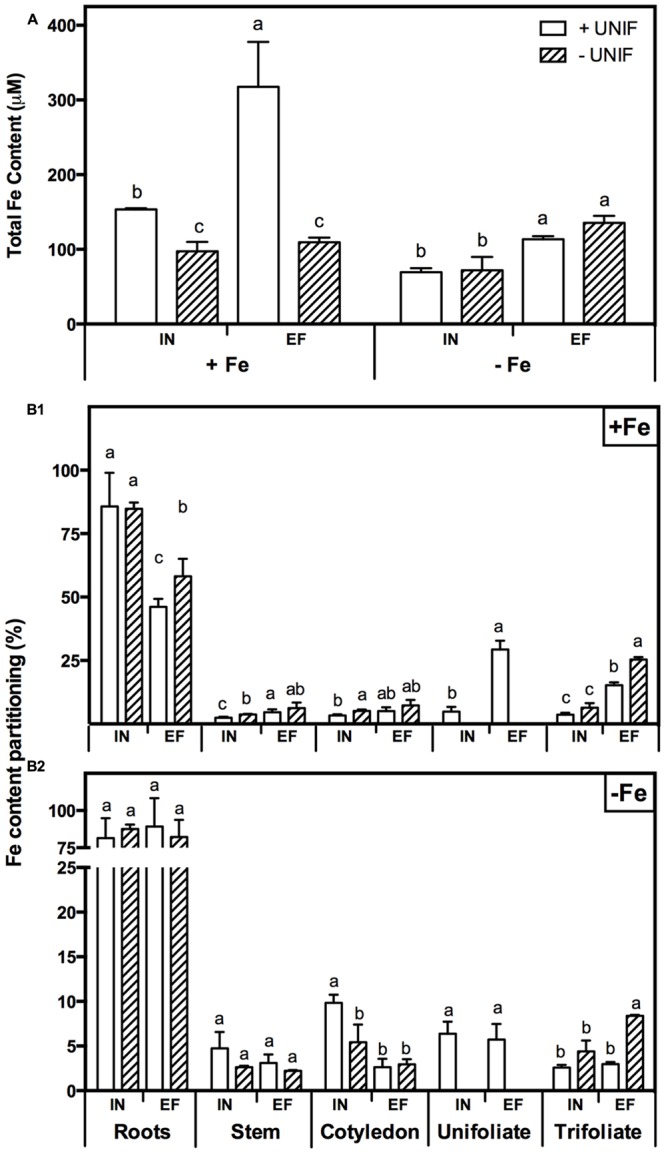
**Iron accumulation profile in two *G. max* accessions [378676A (inefficient) and 437929 (efficient)] grown under Fe-sufficient (+Fe) and Fe-deficient (-Fe) hydroponic conditions, with (+UNIF) and without (-UNIF) unifoliate leaves (Experiment 2): **(A)** Total Fe content, within each Fe treatment different letters indicate significant differences (*P* < 0.05); **(B1)** Fe content percentage (%) in +Fe and **(B2)** Fe content percentage (%) in -Fe, within each plant organ different letters indicate significant differences (*P* < 0.05)**.

In order to study the impact of unifoliate removal on Fe partitioning in each plant organ, IN and EF plants, intact or without unifoliates, were grown under Fe-sufficiency, and Fe-deficiency for 10 days (**Figure 6**, **Table 1**). In Fe sufficient conditions, total Fe content was significantly higher in intact plants (**Figure 6A**). Under Fe deficiency, the removal of the unifoliates had no effect on total Fe content in both accessions, but the EF plants were able to accumulate approximately two times more Fe than the IN plants (**Figure 6A**).

**Table 1:** Fe concentration (μg/g) in root, stem, cotyledon, unifoliate leaves, and trifoliate leaves of inefficient (IN) and efficient (EF) *G. max* accessions grown with (+UNIF) and without (-UNIF) unifoliate leaves, under Fe-deficient (-Fe) and Fe-sufficient (+Fe) hydroponic conditions (Experiment 2).

	Acc. 378676A (IN)				Acc. 437929 (EF)			
	+ Fe		- Fe		+ Fe		- Fe	
	+ UNIF	- UNIF	+ UNIF	- UNIF	+ UNIF	- UNIF	+ UNIF	- UNIF
Root	882 ± 79^b^	1252 ± 46^a^	526 ± 31^c^	816 ± 132^b^	575 ± 37^b^	562 ± 27^b^	655 ± 57^b^	1264 ± 69^a^
Stem	44 ± 1^b^	66 ± 9^a^	48 ± 11^b^	45 ± 1^b^	98 ± 5^a^	76 ± 11^a^	44 ± 9^b^	47 ± 4^b^
Cotyledon	87 ± 13^a^	90 ± 4^a^	90 ± 4^a^	62 ± 9^b^	216 ± 2^a^	108 ± 16^b^	48 ± 12^c^	63 ± 16^bc^
Unifoliate	55 ± 6^a^	-	41 ± 3^a^	-	265 ± 33^a^	-	53 ± 15^b^	-
Trifoliate	96 ± 6^ab^	106 ± 10^a^	61 ± 5^c^	83 ± 1^b^	403 ± 22^a^	243 ± 7^b^	66 ± 4^d^	129 ± 7^c^

Data are the mean of five replicates ± SE. Within each accession and each row different letters indicate significant differences (*P* ≤ 0.05).

The percentage of Fe content of each organ relative to the total Fe content of the whole plant (Fe content partitioning) was calculated to compare the Fe partitioning between plant organs under Fe sufficiency and deficiency (**Figure 6B**). In this case, content was chosen rather than concentration to have a better idea on the total amount of Fe accumulated in one organ in relation to the whole plant accumulation. Looking firstly at intact plants (+UNIF), for both accessions, the organ that had higher Fe concentrations was the root (**Table 1**) and this was also the organ with higher content partitioning (**Figure 6B**). Under Fe sufficiency, the IN plants accumulated higher amounts of Fe in this organ, having about twofold higher Fe content than the EF plants, but under Fe deficiency no significant differences were detected between accessions (**Figure 6B**). The stem was the organ showing lower Fe concentrations amongst all plant organs (**Table 1**), being highest in Fe supplied EF plants. In **Figure 6B** it is also visible that under Fe sufficiency, the EF plants remobilized more Fe to all above-ground organs (stems, cotyledons, and trifoliates) than the IN ones, whilst under Fe deficiency no significant differences were detected between accessions, except in the cotyledons, where the IN plants had higher Fe content partitioning percentages than the EF ones. The trifoliates were the above-ground organ that presented higher Fe concentrations. Under +Fe conditions, the EF plants had a fourfold increase in Fe concentration compared to the IN plants (**Table 1**). As expected, under +Fe conditions, Fe content partitioning was higher in the EF accession (**Figure 6B**).

When looking at the effect of the removal of the unifoliate leaves (-UNIF) it was found that in general unifoliate removal enhanced Fe concentrations in other plant organs (**Table 1**). Under Fe sufficiency, the Fe content partitioning was also enhanced in several instances (**Figure 6B**). Under Fe deficiency, roots of the IN plants accumulated 526 ± 31 ppm Fe and the EF plants 655 ± 57 ppm Fe – whereas by removing the unifoliate leaves, plants accumulated significantly higher amounts of Fe – 816 ± 132 ppm in the IN plants, and 1264 ± 69 ppm for the EF ones (**Table 1**). Also, as plants without unifoliates displayed reduced chlorosis in the trifoliate leaves (**Figures 4A–C**), Fe concentration increased in the trifoliates of IN plants grown under Fe deficiency to similar values of the Fe sufficient intact plants. In the EF plants Fe concentration was two times higher in the trifoliates when unifoliates were removed. Accordingly, the Fe content percentage doubled in the EF trifoliates (**Figure 6B**).

### Fe-Deficiency Related Gene Expression Patterns

**FIGURE 7:**
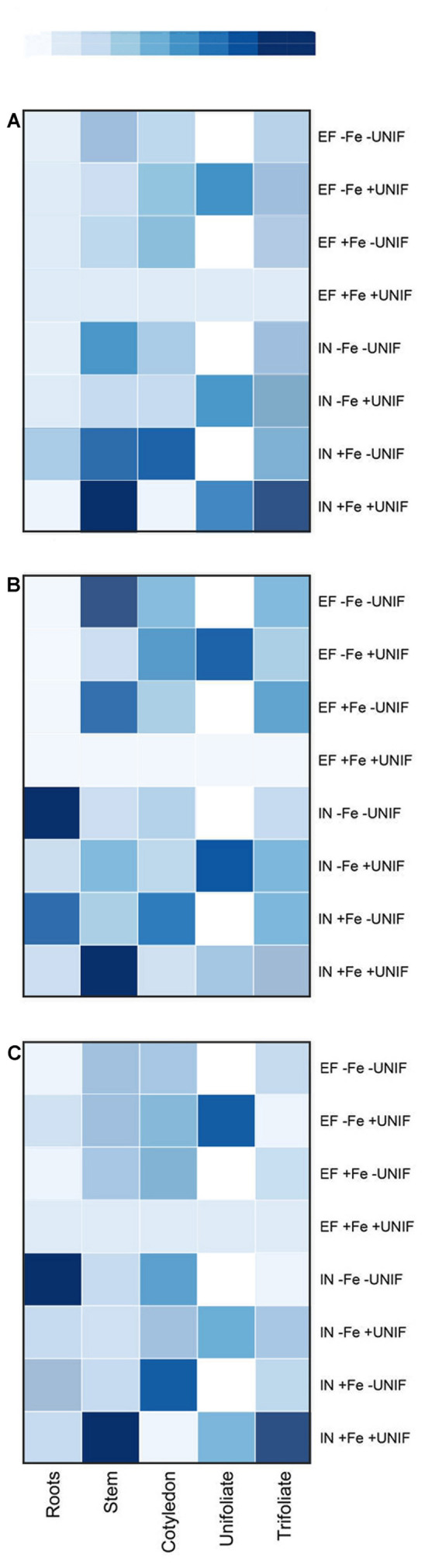
**HeatMap of the expression patterns of *FRO2*-like (A), *IRT1*-like (B) and *ferritin***(C)** genes in roots, stem, cotyledon, unifoliate, and trifoliate leaves of two *G. max* accessions [378676A (IN) and 437929 (EF)] grown under Fe-sufficient (+Fe) and Fe-deficient (-Fe) hydroponic conditions, with (+UNIF) and without (-UNIF) unifoliate leaves (Experiment 2).** “EF +Fe +UNIF” was the reference sample. In dark blue: increased gene expression; in light blue: lower gene expression; in white: unifoliate leaf removed. Total RNA was extracted from a pool of five independent replicates. Corresponding values are presented in Supplementary Tables S3–S5.

Three known genes associated with the Fe-deficiency responses – *FRO2*-like, *IRT1*-like, and *ferritin* – were studied using qPCR. These genes were analyzed separately for each plant organ (**Figure 7**, Supplementary Tables S3–S5) and the impact of unifoliate leaf removal was accessed for both accessions. In general, it was found that regardless of Fe supply, the IN plants presented higher gene expression levels than the EF ones, and that when looking at the expression of these genes in the unifoliate leaves, the expression levels were always higher in the EF plants under Fe-deficiency (**Figure 7**).

Firstly, when looking at the expression of *FRO2*-like (**Figure 7A**), the organ with the lowest expression levels was the root, with variable expression patterns in the remaining plant organs and treatments. Intact EF plants presented low basal levels of *FRO2*-like gene expression in all plant organs. The removal of the unifoliates did not augment the expression of *FRO2*-like gene in the roots of both accessions. In fact, the *FRO2*-like gene here studied appeared to have higher expression levels in the shoots than in the roots.

The expression of *IRT1*-like gene (**Figure 7B**) was higher in the IN plants roots than in the EF ones. After removing the unifoliates, the expression levels in the IN roots was even higher, especially noticeable in plants under Fe deficiency. Although levels of *IRT1*-like gene expression were very low in the EF plants, it had higher levels in the shoots than in the roots, and the expression levels were further increased when unifoliate leaves were removed (**Figure 7B**).

With regards to the *ferritin* gene (**Figure 7C**), the EF plants presented low expression in most plant organs, and the removal of the unifoliates had low impact on the levels of *ferritin* expression. Also, *ferritin* expression levels were similar between Fe sufficient and Fe deficient plants. Contrastingly, in the IN plants, the expression was highest in the stems and in the trifoliate leaves of Fe-sufficient plants, and the removal of the unifoliate leaves decreased the expression levels in these organs (**Figure 7C**). Interestingly, *ferritin* expression was higher in the roots of the plants without unifoliate leaves than in the intact plants, and these plants were the ones that accumulated more Fe (**Figure 6**).

## Discussion

In the current work the efficiency trait was associated with lower chlorosis development under Fe deficiency (**Figure 2**) according to what has been previously described (Brown, 1978). Fe is essential in redox reactions, often used in electron transport chains, as well as in metabolic processes. Chlorophyll biosynthesis requires Fe, and plants need concentrations of 10^-9^ to 10^-4^ M to achieve optimal growth (Kim and Guerinot, 2007). Also, root Fe reductase is known to be the rate-limiting enzyme for Fe uptake (Wu et al., 2012; García et al., 2013). The ferric reductase activity was analyzed in roots of the IN and EF plants, to confirm if under Fe deficiency the EF plants had higher reductase activity as the efficiency trait has been associated with a reductase activity inducible by Fe deficiency (Moog and Brüggemann, 1994). However, here, for both IN and EF plants, the enzyme was more active in Fe-sufficient conditions than in Fe deficiency (**Figure 3**). It is well-known that Fe reductase activity is usually induced under Fe deficiency (Vert et al., 2003; Kong et al., 2013; Wang et al., 2013; Zha et al., 2014). However, in the current work, we observed that under Fe restriction plants had lower reductase activity than under Fe sufficiency. In fact, this is an observation that was already made before in Williams 82 soybean lines (Santos et al., 2013), and was also registered in common bean (Blair et al., 2010). In fact, it seems that reductase induction is not only species dependent (Santos et al., 2013), but also cultivar dependent (Blair et al., 2010; Pereira et al., 2014). These findings could be related to the fact that the Fe reductase enzyme itself has a heme containing Fe group. Thus, having grown the plants in total absence of Fe may have impaired the synthesis or functioning of the enzyme, which could explain the low reduction values in plants grown under Fe deficiency.

Moreover, although the EF plants had higher values of reductase activity under Fe-sufficiency than the IN ones, this alone cannot explain the difference in the efficiency, since under stress conditions none of them were able to activate the enzyme.

### Unifoliate Leaf Removal Reduced Chlorosis and Increased Shoot to Root Ratio

As young leaves are one of the major sinks during the early developmental stages of plant growth and the access to photoassimilates and nutrients must be balanced between sinks (Wardlaw, 1990), we hypothesized that the removal of the unifoliate leaves could alleviate chlorosis and other IDC symptoms, since the competition between sinks would decrease. To this end, the unifoliate leaves were removed at a very young stage (in **Figure 1** the morphology of a soybean plant at an early growth stage is depicted) to understand if Fe would be directed to other sinks, diminishing the stress from Fe deprivation. This hypothesis seems to be supported by the observation that soybean plants with bigger unifoliates are more IDC susceptible (Vasconcelos and Grusak, 2014). In fact, under Fe-deficiency, unifoliate removal led to a visual reduction of chlorosis (**Figure 4A**) and a concomitant increase in SPAD values (**Figure 4C**), particularly in the EF plants. The fact that the IN plants when grown without unifoliate leaves (a strong sink for Fe) still showed a certain degree of chlorosis reveals that other processes are limiting Fe availability at leaf level (e.g., the level of remobilization, the storage form of Fe in source tissues, the amount of chelators, the type and amount of organic acid release or the expression of specific transporters).

Our results show that under Fe-deficiency the EF plants had higher root DW than the IN ones (**Figure 5B**), while the shoot DW did not vary among accessions (**Figure 5A**). This increased root to shoot DW ratio reflects the ability of the EF plants to allocate more resources to the organs involved in mineral acquisition when under shortage of mineral nutrients (Marschner et al., 1996; Hermans et al., 2006; Lemoine et al., 2013). Additionally, in plants without unifoliate leaves, root DW decreased whereas shoot DW increased, and they did not differ between accessions. This investment in the aerial organs rather than on the roots is possibly due to a lower sink demand and therefore a diminished Fe-deprivation stress.

### Efficient Plants were able to Better Translocate Fe to the Trifoliate Leaves

The EF and IN plants responded differently in terms of Fe accumulation and distribution. When under Fe-deficiency (with or without unifoliate leaves), the EF plants had higher total Fe content (**Figure 6A**). Roots were the organs that accumulated more Fe (**Figure 6B**, **Table 1**). Moreover, under Fe-deficiency, roots of plants without unifoliates accumulated more Fe than roots of the intact plants and no differences were detected in Fe content percentage between accessions. Under optimal, Fe sufficient conditions, the EF plants had lower Fe content in the roots than the IN plants, indicating that less Fe was retained in this organ and was possibly distributed along the plant aerial organs. Unifoliate leaf removal led to an increase in the Fe concentration in the roots of the IN plants, but not in the EF plants (**Table 1**), suggesting that the IN plants have an impairment of Fe re-distribution.

The trifoliate leaves of plants without unifoliates, which were the ones with higher SPAD values and lower IDC visual scores (**Figure 4**), had higher Fe concentrations and Fe content percentages, especially in the EF plants. Studies show that higher Fe concentrations can be found in young chlorotic leaves, when compared to green leaves, the so called “chlorosis paradox” that can result from an Fe inactivation in the plant under alkaline conditions (Römheld, 2000). However, in more recent works plants under Fe deficiency have lower Fe concentration in the leaves, showing that this is not an ubiquitous phenomenon, both in hydroponic (Ramírez et al., 2013; Kong et al., 2014), and soil conditions (Chakraborty et al., 2013; El-Jendoubi et al., 2014). Additionally, studies on Fe partitioning show that under Fe-deficiency and sufficiency the senescence of older leaves with a reduction of their sink capacity results in Fe retranslocation to younger leaves (Shi et al., 2012). Here, the removal of the unifoliate leaves also led to an Fe translocation toward the young trifoliate leaves of both IN and EF plants, specially under Fe-deficiency (**Table 1**, **Figure 6B**).

The EF plants grown under Fe-sufficiency, regardless of having unifoliates or not, were the ones with lower Fe concentration in the roots and had a more balanced distribution of the Fe pools throughout all organs, resulting in higher concentrations accumulating in the trifoliate leaves (**Table 1**, **Figure 6B**). There are several theories behind Fe deficiency sensing, but no consensus was yet achieved (for a recent review please see García-Mina et al., 2013). Roots were firstly proposed as the main organ for Fe-deficiency sensing (Bienfait et al., 1987) but more recent research shows that shoots have an important role in the regulation of the Fe-stress signaling (Enomoto et al., 2007; Wu et al., 2012). Our data suggests that the ability to translocate mineral resources from root to shoot contributes directly to the plants’ efficiency trait.

### The IN Accession Presented Enhanced Gene Expression Levels

In the current work, the IN plants had higher values of gene expression. These results were also obtained in other studies, where soybean inefficient lines responded to Fe stress by increasing the transcripts of genes involved in, for example, signaling and hormonal regulation (O’Rourke et al., 2007). The EF accession, not having suffered as severely as the IN one to the Fe shortage, it did not have the necessity to trigger Fe-uptake related genes. Besides the generally higher levels of gene expression by the IN accession, differences in the expression of individual genes were also apparent.

As previously discussed, the activity of the root reductase was very low under Fe-deficient conditions (**Figure 3**). At a molecular level, the expression of *FRO2*-like gene was also very low in the roots, independent of the growth conditions. A time-course study in tomato showed that the activation of Fe deficiency stress response occurs in a progressive way, reaching a peak 5 days after Fe depletion and decreasing afterward; also, it showed that the variation in FRO1 transcript level is directly proportional to the root ferric chelate reductase activity (Paolacci et al., 2014).

Additionally, a strong induction of this gene was detected in the shoots, and the removal of the unifoliate leaves led to an increase of the expression under Fe-deficiency. As aforementioned, Fe must be reduced from Fe(III) to Fe(II) at the root surface for the uptake process. However, after entering across the rhizodermal plasma membrane barrier, it is again oxidized and transported through the xylem as an Fe(III) citrate complex, and for assimilation in leaves Fe must be again reduced (Wu et al., 2005). It is possible that the expression of *FRO2*-like in the shoot was increased to free the unavailable Fe (III) and make it more accessible for distribution within the plant. On the other hand, *FRO2* is a member of a gene family that comprises eight members in *Arabidopsis*, each one with tissue specificity (Wu et al., 2005); probably the *FRO* gene here analyzed is not the principal root reductase in soybean, but is functionally more similar to *AtFRO7*, which is known to be more active in the shoots of *Arabidopsis thaliana* (Jeong et al., 2008) than in the roots.

After the reduction step, *IRT1* is necessary for Fe transport, and a strong induction of *IRT1*-like gene expression was detected in the IN plants’ roots after the removal of the unifoliates (**Figure 7B**). *IRT1* is usually up-regulated in Fe-deficient conditions, but studies show that its regulation is dependent both on the root Fe pool and on the shoot Fe demand (Vert et al., 2003). Our results also show a strong induction of *IRT1*-like gene in the EF plants shoots (**Figure 7B**). *IRT1* belongs to a family of genes – ZIPs – detected in different tissues (roots, leaves, nodules, and flowers) and it may also be involved in the transport pathways to other plant organs (Grotz et al., 1998). It has been shown that some members of the *ZIP* family could be associated not only with Fe uptake, but also with detoxification and storage of excessive Fe (Yang et al., 2009; Li et al., 2013). This could be the case in the EF plants, since as the unifoliate leaves were removed, more Fe was accumulated in the aboveground organs (**Table 1**, **Figure 6**), and *IRT1*-like gene expression was increased, corroborating its role in Fe homeostasis maintenance.

Ferritins are encoded by nuclear genes regulated by Fe and store Fe in its oxidized form (Harrison and Arosio, 1996). The IN accession had higher induction levels of the *ferritin* gene than the EF one. It has been suggested that Fe, when stored in the form of ferritin, may not be readily available for retranslocation (Vasconcelos and Grusak, 2014). It is possible that the higher accumulation of Fe with ferritin by the IN plants could be responsible for its lower partitioning capacity (**Table 1**, **Figure 6B**). Alternatively, the induction of ferritin synthesis is correlated with the degree of PSI degradation during Fe deficiency (Briat et al., 2010), which is in accordance to the results presented here: IN plants, that had acuter chlorosis symptoms and, therefore, higher degradation of PSI, had a strong induction of the expression in the trifoliate leaves (**Figure 7C**). With the removal of the unifoliate leaves, the levels of *ferritin* gene expression were lowered in the trifoliates, and so did the symptoms of chlorosis (**Figure 4**), again confirming that Fe bound to ferritin in the roots could have been hampering its partitioning to the aerial parts.

## Conclusion

Although it is known that Fe deficiency induces both morphological and physiological responses in plants (Wu et al., 2012), how these responses are triggered is still unclear. Moreover, the partitioning of Fe between different plant organs could be a key mechanism for plant adaptation to this type of stress and could be related to the expression of specific genes.

Our results corroborated our initial hypothesis that the ability to remobilize Fe could be related to IDC susceptibility. The removal of the unifoliate leaves increased total Fe content in Fe-deficient conditions but Fe was mainly accumulated in the roots. Nonetheless, in the EF accession without unifoliate leaves, Fe concentration also significantly augmented in the trifoliate leaves and IDC symptoms were alleviated almost to full correction. Moreover, the EF plants under optimal conditions were able to distribute Fe in a more balanced way throughout all organs, fact that was not verified in the IN plants, suggesting that the ability to translocate Fe from the roots to the aboveground organs could explain the different IDC susceptibility between accessions.

Moreover, the IN plants induced higher expression levels of Fe uptake related genes, which may be an indicator of the higher susceptibility of this accession to Fe deficiency and shows that low gene expression levels cannot be responsible for the plant’s low efficiency, as previously suggested (O’Rourke et al., 2007). The high level of ferritin expression by the roots of the IN plants could be responsible for the accumulation of Fe with ferritin in this plant organ and, consequently, making Fe partitioning to the shoots more difficult.

The enhanced overall growth of the plant and the reduced chlorosis and Fe accumulation in the trifoliate leaves here obtained by the removal of the unifoliate leaves appears to be due to a reduction in source–sink imbalance that reduced IDC symptoms. These findings suggest a key role of shoots in Fe-stress response signaling and identified possible factors that could influence plant IDC susceptibility. This comprehensive analysis helped to better understand some of the mechanisms behind mineral partitioning and resource allocation in soybean, and our conclusions can possibly be extrapolated to other agricultural crops suffering from IDC. Still, nutrient solutions cannot fully mimic agricultural, alkaline soil conditions, and as such further studies are necessary to extrapolate our findings to natural settings.

## Author Contributions

CS carried out the sample preparation and analysis, gene confirmation experiments and drafted the manuscript; MR performed the ICP-OES analysis and helped in results interpretation; SC helped conceive the study and its design, and participated in the critical review of the manuscript; MV conceived the study, its design and coordination and helped to draft the manuscript. All authors read and approved the final manuscript.

## Conflict of Interest Statement

The authors declare that the research was conducted in the absence of any commercial or financial relationships that could be construed as a potential conflict of interest.

## Footnotes

1 http://www.ars-grin.gov/

2 Frodo.wi.mit.edu

3 www.graphpad.com

## Abbreviations

DW: dry weight
EF: Fe-efficient
ICP-OES: inductively coupled plasma optical emission spectrometer
IDC: iron deficiency chlorosis
IN: Fe-inefficient
PQ: partition quotient
SEM: standard error of mean.
