# Supplementary material for: Iron partitioning at an early growth stage impacts iron deficiency responses in soybean plants (Glycine max L.)
Source: Front Plant Sci. 2015 May 12;6:325. doi: 10.3389/fpls.2015.00325 (PMC4428275; doi:10.3389/fpls.2015.00325)
Supplement: Supplementary file 1 [file Table_1.DOCX]

**Table S1.** Sequence orthologs of *Arabidopsis thaliana* *FRO2* and *IRT1* genes in *Glycine max*

| Gene | *G. max*  Accession nr. | *A. thaliana*  Accession nr. | Maxim score | Total score | Query cover | E value | Identity |
| --- | --- | --- | --- | --- | --- | --- | --- |
| *FRO2-*like | XM_003548612.1 | NM_100040.2 | 399 | 399 | 91 % | 5e^-109^ | 65 % |
| *IRT1-*like | XM_003520096.2 | NM_118089.3 | 105 | 105 | 19 % | 9e^-21^ | 69 % |

**Table S2.** Primer sequences and correspondent accession numbers (Acc. No)

| Primer | Forward (5’-3’) | Reverse (5’-3’) | Acc. No |
| --- | --- | --- | --- |
| *18S* | TTAGGCCATGGAGGTTTGAG | GAGTTGATGACACGCGCTTA | X75080.1 |
| *FRO2*-like | TGCTTGGACTCACACCAGAG | AGAGGTAGAAACCGGGGAGA | XM_003548612.1 |
| *IRT1*-like | GATTGCACCTGTGACACAAA | CAGCAAAGGCCTTAACCATA | XM_003520096.2 |
| *Ferritin* | CCCCTTATGCCTCTTTCCTC | GCTTTTCAGCGTGCTCTCTT | U31648.1 |

**Table S3.** *FRO2* gene RNA relative expression values of inefficient (IN) and efficient (EF) *G. max* plants grown under Fe-sufficient (+Fe) and Fe-deficient (-Fe) hydroponic conditions, with (+UNIF) and without (-UNIF) unifoliate leaves

| Treatment | Roots | Stem | Cotyledon | Unifoliate | Trifoliate |
| --- | --- | --- | --- | --- | --- |
| IN +Fe +UNIF | 0.140 | 3054 | 0.849 | 3.41 | 24.3 |
| IN +Fe -UNIF | 1.75 | 4.40 | 3.71 | -- | 6.50 |
| IN -Fe +UNIF | 0.226 | 0.977 | 1.75 | 2.80 | 6.45 |
| IN -Fe -UNIF | 0.250 | 3.35 | 2.93 | -- | 4.39 |
| EF +Fe +UNIF | 1.00 | 1.00 | 1.00 | 1.00 | 1.00 |
| EF +Fe -UNIF | 0.258 | 1.33 | 2.24 | -- | 3.05 |
| EF -Fe +UNIF | 0.863 | 1.17 | 2.36 | 2.83 | 3.87 |
| EF -Fe -UNIF | 0.627 | 3.38 | 1.76 | -- | 3.02 |

**Table S4.** *IRT1* gene RNA relative expression values of inefficient (IN) and efficient (EF) *G. max* plants grown under Fe-sufficient (+Fe) and Fe-deficient (-Fe) hydroponic conditions, with (+UNIF) and without (-UNIF) unifoliate leaves

| Treatment | Roots | Stem | Cotyledon | Unifoliate | Trifoliate |
| --- | --- | --- | --- | --- | --- |
| IN +Fe +UNIF | 0.998 | 835 | 0.953 | 3.51 | 12.0 |
| IN +Fe -UNIF | 5.76 | 2.86 | 3.07 | -- | 2.95 |
| IN -Fe +UNIF | 1.16 | 2.72 | 1.95 | 3.66 | 2.51 |
| IN -Fe -UNIF | 186 | 1.40 | 2.30 | -- | 2.68 |
| EF +Fe +UNIF | 1.00 | 1.00 | 1.00 | 1.00 | 1.00 |
| EF +Fe -UNIF | 0.538 | 2.35 | 1.23 | -- | 1.59 |
| EF -Fe +UNIF | 1.03 | 1.53 | 2.30 | 2.53 | 1.62 |
| EF -Fe -UNIF | 0.538 | 3.60 | 1.38 | -- | 1.45 |

**Table S5.** *Ferritin* gene RNA relative expression values of inefficient (IN) and efficient (EF) *G. max* plants grown under Fe-sufficient (+Fe) and Fe-deficient (-Fe) hydroponic conditions, with (+UNIF) and without (-UNIF) unifoliate leaves

| Treatment | Roots | Stem | Cotyledon | Unifoliate | Trifoliate |
| --- | --- | --- | --- | --- | --- |
| IN +Fe +UNIF | 0.705 | 663 | 0.849 | 1.18 | 6.23 |
| IN +Fe -UNIF | 5.55 | 0.883 | 3.71 | -- | 1.49 |
| IN -Fe +UNIF | 0.964 | 0.777 | 1.75 | 1.27 | 1.64 |
| IN -Fe -UNIF | 188 | 1.24 | 2.93 | -- | 1.82 |
| EF +Fe +UNIF | 1.00 | 1.00 | 1.00 | 1.00 | 1.00 |
| EF +Fe -UNIF | 0.582 | 1.84 | 2.24 | -- | 1.27 |
| EF -Fe +UNIF | 1.35 | 2.08 | 2.36 | 2.189 | 1.79 |
| EF -Fe -UNIF | 0.520 | 1.97 | 1.76 | -- | 1.08 |
